# Retrograde Percutaneous Release of Trigger Finger or Thumb Using Sono-Instruments®: Detailed Technique, Pearls, and Pitfalls

**DOI:** 10.7759/cureus.52911

**Published:** 2024-01-25

**Authors:** Fabian Moungondo, Luc Van Ovestraeten, Mohammad O Boushnak, Frédéric Schuind

**Affiliations:** 1 Department of Orthopedics and Traumatology, Université Libre de Bruxelles, Erasme University Hospital, Brussels, BEL; 2 Department of Orthopaedics and Traumatology, Hand and Wrist Center, Hand and Foot Surgery Unit (HFSU), AO Foundation, Erasme University Hospital, Tournai, BEL; 3 Department of Orthopedics and Sports Medicine, North Sydney Orthopaedic and Sports Medicine Centre, Mater Hospital, Sydney, AUS

**Keywords:** diabetic patients, outpatient clinic, percutaneous surgery, percutaneous trigger finger release, sono-instrument

## Abstract

Percutaneous release is a common treatment option for trigger finger stenosing tenosynovitis. While surgical and conservative treatments are available, percutaneous techniques offer several advantages, including faster recovery time, reduced complications, and simultaneous treatment of multiple trigger fingers. The sono-instrument is a minimally invasive device designed for surgical release of the A1 pulley in adults. The device is efficient and safe, and in addition, several design features enhance the visibility of the instrument under ultrasound imaging. The technique is truly percutaneous, as the whole operation is done through a single needle puncture. This minimizes postoperative discomfort and allows an immediate return to daily living and professional activities. The technique can be performed in an outpatient clinic under local anesthesia. The learning curve is quick; however, surgeons must acquire experience in hand sonography to master this new form of surgery. The aim of this article is to provide an in-depth exposition of the technical nuances, pearls, and pitfalls of this novel retrograde percutaneous release method. To our knowledge, this is the first retrograde truly percutaneous release technique yet described, facilitated by the novel Sono-Instruments®.

## Introduction

Stenosing tenosynovitis is a common condition affecting 2% of the general population [1]. It is usually caused by thickening of the A1 annular pulley, thereby leading to pain, decreased range of motion, triggering, and swelling [2,3]. Both conservative and surgical methods of treatment are described in the literature, with a rate of success of 50%-92% and 100%, respectively [3,4].

Percutaneous release of the A1 digital pulley is an alternative to the traditional open technique [3]. The potential advantages of percutaneous techniques are fewer complications, faster recovery time, and the ability to treat multiple trigger fingers simultaneously without multiple scars or post-operative stiffness [1,5]. Percutaneous techniques were first introduced by Lorthioir et al. in 1958, who proposed blind release of the A1 pulley [6]. It has since been improved with the introduction of ultrasound guidance, which allows clear identification of anatomical structures surrounding the A1 pulley and of the pulley itself, thereby decreasing the risk of complications related to neurovascular and flexor tendons injury [7-10]. The optimal device to perform percutaneous release is still a matter of debate. Several authors have reported acceptable outcomes using 19-21-gauge needles, with a success rate reaching 81.7% [8,11]. However, sharp-tip needles put surrounding structures at risk of injury, while small-sized needles are predisposed to bending and twisting, which can lead to incomplete release and tendon laceration [8,11]. Other authors proposed the use of first-generation hook knives that may be used to cut from outside to inside the A1 pulley (“extra-sheath” release) or from inside to outside the A1 pulley (“intra-sheath” release) [12]. These techniques have drawbacks, including the sharpness of the hook, which exposes them to iatrogenic injuries. Second-generation knives are also available to the surgeon, including minimally invasive endoscopic surgical knives first designed for carpal tunnel release and later adapted to the fingers and other knives designed specifically for the trigger finger [11-13]. These knives present a relatively straight design, which makes them difficult to use and limits their accuracy. A skin incision in the palm is needed to dissect the pulley and introduce the knife. When used for the thumb trigger finger, these devices are especially dangerous for the radial digital nerve, where instead open release is usually recommended [13].

Recently, a new device has become available for percutaneous retrograde A1 pulley release for trigger finger treatment, the Trigger-Finger Sono-Instrument® (TF-SI) (Spirecut SA, Hofackerstrasse 40B, CH-4132 Muttenz, Switzerland) [14,15]. This instrument, with a diameter of 1.5mm, allows for the operation through a simple needle puncture. The general shape of the instrument is adapted to the adult flexor digital sheath anatomy, allowing for safe cutting of the A1 (and, if needed, the A0) annular digital pulley(s). It is effective on tense ligament fibers but not sharp enough to inadvertently injure tendons, vessels, or nerves. A premarket clinical prospective study conducted by the authors and accepted for publication using this device has proven its safety and efficiency in a small series of 15 trigger fingers or thumbs [15]. Despite its emerging use for trigger finger release, there is yet no published standardized technique for employing this retrograde approach. The aim of this study was to develop and describe a standardized method for using the TF-SI in a retrograde percutaneous release of trigger fingers or thumbs, detailing the essential steps, nuances, and considerations crucial for successful outcomes.

## Technical report

Ultrasound equipment and TF Sono-instrument design

**Figure 1:**
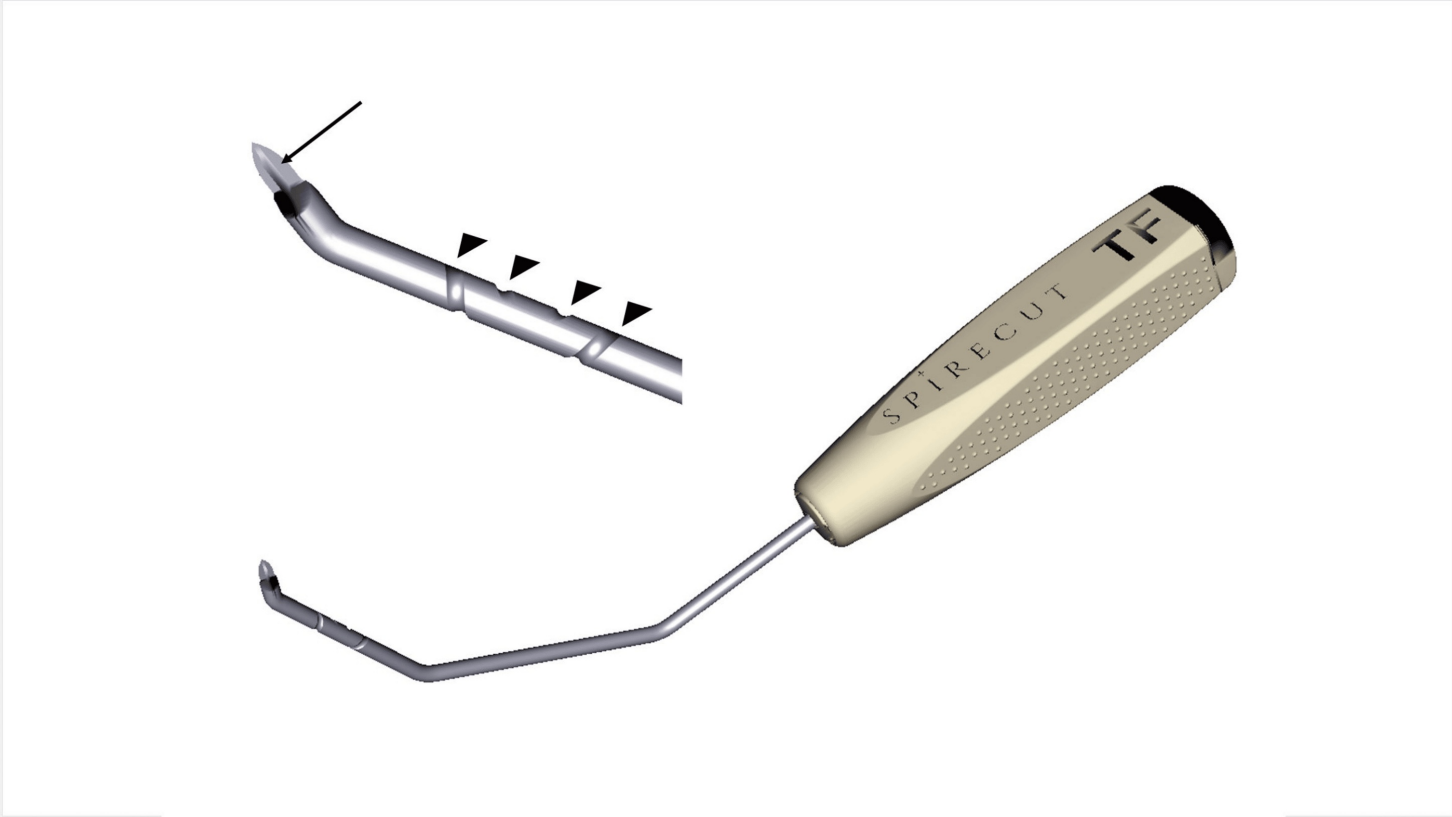
Trigger finger Sono Instrument® features The TF-SI presents an angulated, sharp end that allows for an A1 pulley progressive cut. A specific flange is added on the unbeveled side of the tip to enhance the sonographic visualization of the cutting part (arrow). A spiral groove on the instrument rod allows for the identification on the sonography image of the amount of rotation of the instrument (arrow heads). Image created by F Moungondo

With the digital pulleys being a few millimeters under the skin, the sonograph used to guide percutaneous release needs to provide accurate imaging of superficial tissues. This is possible with modern machines and high-frequency (> 12 MHz) linear probes. A high-frequency hockey stick probe is particularly convenient for trigger finger surgery, as bigger sonography probes may be in the way of the percutaneous operation. The whole operation is done in B-mode. The TF-sono-instrument has special features (lateral flange reflecting echoes at the cutting extremity and a spiral groove on the rod) that have been designed to enhance ultrasound visualization while also allowing rotatory orientation (Figure 1).

Anatomy

**Figure 2:**
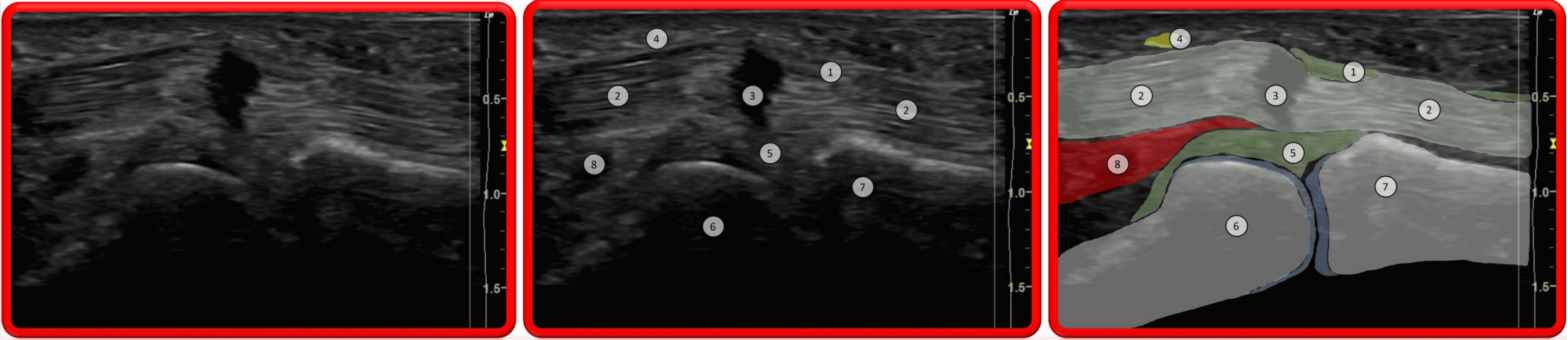
Trigger thumb sonogram Longitudinal view: 1) A1 annular pulley; 2) FPL; 3) FPL anisotropic artifact (frequently observed in cases of triggering with impaired tendon excursion); 4) Thumb radial digital nerve; 5) Palmar plate; 6) Head of first metacarpal; 7) Base of proximal phalanx; 8) FPB. Image taken from the studied patient.

**Figure 3:**
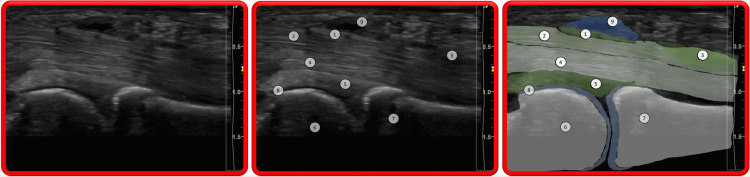
Trigger digit (long finger) somogram Longitudinal view: 1) Thickened A1 annular pulley; 2) FDS; 3) Nodule within FDS; 4) FDP; 5) Palmar plate; 6) Head of first metacarpal; 7) Base of proximal phalanx; 8) Hyaline cartilage of head of metacarpal bone; 9) Ganglion on A1 annular pulley. Image taken from the studied patient.

A perfect understanding of the surgical and ultrasonographic anatomy is of paramount importance to performing ultrasound-guided A1 pulley release (Figures 2, 3). The metacarpo-phalangeal joint is the osteo-articular landmark, allowing one to locate the flexor tendon(s) of the affected finger or thumb and the annular A1 digital pulley. The metacarpal head-neck junction and the proximal phalangeal base-shaft junction are the relevant bony landmarks to locate the proximal and distal A1 pulley edges, respectively [12]. It is crucial to observe the gliding of the flexor tendon(s) during passive and active motion (sagittal, also called long axis view) and to clearly identify the digital neuro-vascular bundles to be preserved (better observed in transverse, also called short axis view).

Long Fingers

The A1 annular pulley is a fibrous structure at the base of the finger that prevents flexor tendons from bowstringing. The sonographic appearance of the A1 pulley has been studied by Guerini et al. [16]. In long-axis view, passive motion of the finger facilitates the identification of the pulley as a thickened tissue, usually flat on the sheath side and convex superficially, not moving with the tendons, and extending from the level of the metaphysis of the metacarpal bone to the level of the metaphysis of the proximal phalanx. In short-axis view, the pulley is well visible at the level of the joint (where no bone is seen under sonography). In this view, the pulsating digital arteries are easy to identify, allowing us to find the digital nerve close by. The flexor tendons are well visible in long-axis and short-axis views. Dynamically, the superficialis and profundus tendons move together with passive or active global digital motion.

Thumb

Because of the anteposed position of the thumb, the installation for sonography is less easy. The thumb should be held by an assistant in metacarpo-phalangeal extension. The metacarpo-phalangeal joint allows for the identification of the A1 pulley. In the long-axis view, the appearance of the joint at the level of the pulley is different than for a long finger. For the long finger, the volar plate joins the metacarpal bone to the phalanx; for the thumb, one sesamoid bone is also seen. It is crucial to well identify the digital nerves, particularly the radial digital nerve, which crosses obliquely the flexor digital sheath at some distance, proximal to the A1 pulley, but sometimes quite close [20]. Fortunately, this nerve is thick and easily identified in the long-axis view. The nerve is sometimes quite mobile and can be pushed by the sonography probe in the field of percutaneous release, representing a danger of an iatrogenic lesion. The surgeon should pay attention to this possibility.

Pathological Abnormalities 

Ultrasound is usually used to visualize the cause of triggering and/or impaired finger or thumb motion. Typically, but not always, the A1 annular pulley is thickened (normal: < 0.6mm); the passive and especially active motion of the flexor tendon(s) is irregular; there is asynchronous motion between the superficialis and the profundus tendons in long fingers [16]; a tendon node can sometimes be observed; an anisotropy artifact can be seen in the tendon(s) stuck by the thickened A1 pulley due to the angulation of tendon fibers induced at this location; there is sometimes fluid or synovitis around the tendons, not to be confused with the anisotropy artifact (“Mona Lisa" image) seen in short axis view from either side of the thickened digital pulley. [16] Occasionally, there is an associated digital sheath ganglion. The pathological abnormalities are shown in Figures 2, 3.

Indication and contra-indications

Percutaneous A1 release using a sono-instrument is indicated for adult stenosing tenosynovitis with typical signs and symptoms. It is also indicated for patients with confirmed painful altered flexor tendon(s) gliding and/or increased thickness of the A1 digital pulley under sonography. A relative contra-indication is a situation with a longstanding flexion contracture of the proximal interphalangeal joint, sometimes best treated by resection of a superficialis slip. Known allergies to local anesthetics and metals are contraindications for sono-instrument surgery.

Technique

Setup and Anesthesia

The patient is positioned in a supine position for the procedure. No tourniquet is used. The surgeon uses the dominant hand to hold the sono-instrument and the non-dominant hand to hold the sonography probe, and this determines if he/she is sitting close to the head or close to the trunk of the patient. A pre-operative sonography is performed to investigate statically and dynamically the surrounding structures anatomy, making sure that there is no normal or pathological condition contra-indicating the operation, and to verify under non-sterile conditions the general installation of the patient and of the sonography probe and screen for the comfort of the patient, to evaluate the feasibility of the operation under local anesthesia, to assess the cooperation of the patient, and to assess the technical ease for the surgeon.

The limb is then disinfected and draped. The necessary sterile material includes the TF-SI, a 10ml syringe with a 23-gauge, 25-mm needle for local anesthesia, a 14-gauge intravenous catheter to perforate the skin and the flexor tendon sheath, sterile sonographic gel, a sterile transparent cover for the sonographic probe, and sponges and material for the compressive dressing at the end of the operation. The sterile transparent cover is disposed around the hockey stick sonographic probe with some gel on it, avoiding any space containing air between the probe and the sterile transparent cover. A support allowing you to hold the hand may be helpful in maintaining the finger metacarpo-phalangeal joint in slight hyperextension. Alternatively, thick sponges may be used to provide this support. For the thumb, the assistant holds the metacarpo-phalangeal joint in full extension.

**Figure 4:**
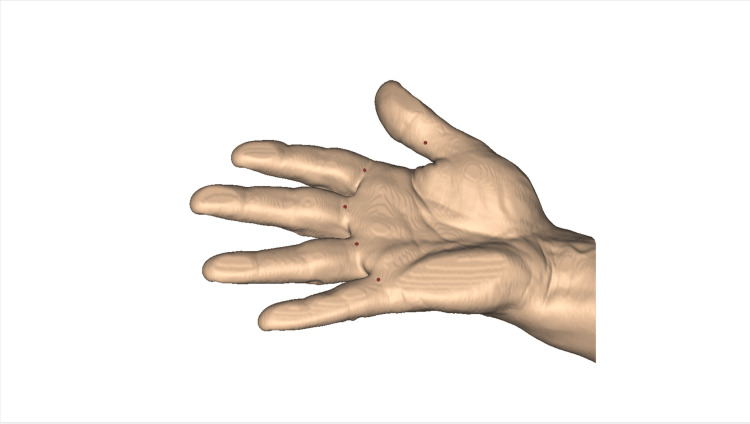
Entry point location (red dots) depending on finger A1 pulley to release Note that the entry point is located at the metacarpo-phalangeal flexion crease for long fingers and more distal, at the middle of the proximal phalanx, for the thumb. Image created by F Moungondo.

The surgeon repeats the pre-operative sonographic imaging of the finger, now under sterile conditions, identifying again important landmarks (metacarpo-phalangeal joint, palmar surfaces of metacarpal and proximal phalanx, palmar plate), the structures at risk (in particular, the neurovascular intermetacarpal and digital bundles), the flexor tendon(s), and the tissue to be released, usually the A1 annular pulley, sometimes also the A0 pulley, in short axis and long axis planes. A dynamic evaluation of tendon gliding and joint motion is performed under sonography. It is frequent to observe the phenomenon of triggering or altered gliding of the flexor tendons relative to each other or to the sheath. There may be abnormalities in the structure of the tendons themselves. The optimal entry point for local anesthesia and, subsequently, for the operation of percutaneous release is then determined. It is volarly in the finger midline, for the long fingers, at the metacarpo-phalangeal crease, or slightly more proximal. It is slightly ulnar for the index and radial for the little finger, in line with the axis of the flexor tendons. For the thumb, the optimal entry point is at the mid-proximal phalanx (Figure 4).

**Figure 5:**
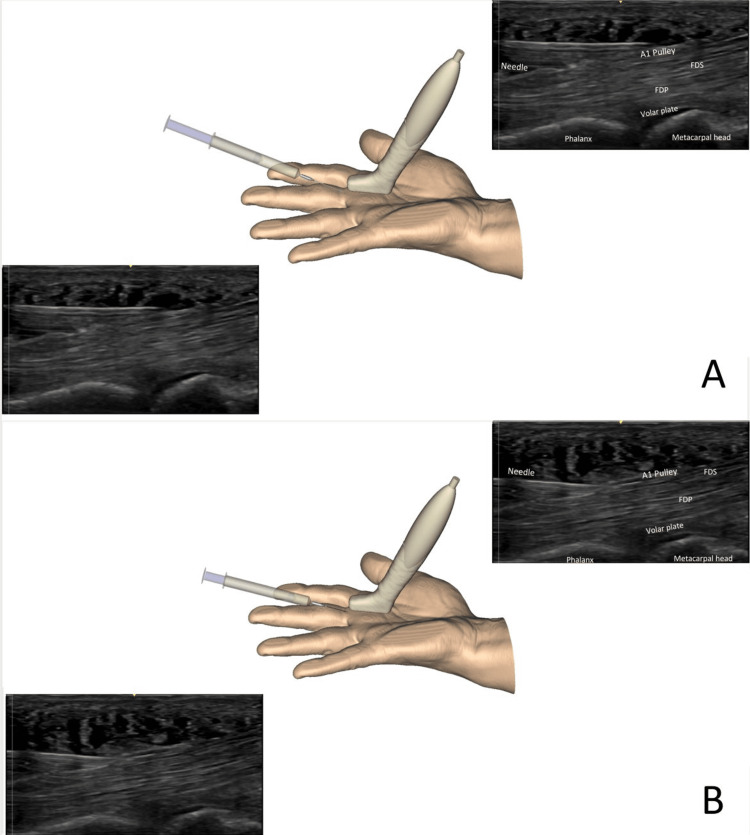
Local anesthesia injection technique Injection is performed over the A1 pulley (A) and into the volar sheath (B). To better understand the sonographic view, the image on the left and bottom is repeated right on top, with a legend. FDS: Flexor digitorum superficialis, FDP: Flexor digitorum profundus Image created by F Moungondo.

At the entry point, the surgeon proceeds to local anesthesia (lidocaine 1% without adrenalin), under sonographic guidance, from distal to proximal, subcutaneously, superficial to the flexor digital sheath, then deep to the annular pulley (Figure 5). The surgeon holds the syringe with one hand, ready to inject, with the needle bent and the bevel facing the deep tissues. The injection usually allows transiently better visualization of the pulley. Care is taken to avoid the injection of air, which would alter sonographic imaging. The injection might be moderately painful, especially when performed into the flexor sheath; the patient should be averted of it just before this injection to avoid any unwanted movement. New dynamic sonographic imaging is then obtained, detecting subtle friction or triggering phenomena not seen before the anesthesia, as active finger motion is now painless.

Opening the Flexor Tendon Sheath

As the TF-SI is not a perforating device, the 14-gauge catheter, held very horizontally, is now inserted through the skin at the entry point, then through the tendon sheath, perforated just distal to the A1 pulley. The tip of the catheter is accurately identified by slight rotations of its bevel. The authors don’t routinely use the probe provided by the manufacturer; it is usually too thick to be placed in the tight tendon sheath.

Percutaneous Pulley Release

**Figure 6:**
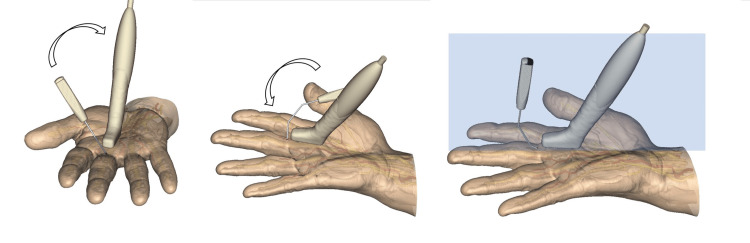
Introduction of the TF-SI The instrument is introduced quite horizontally at first. Once the tip is seen under sonography, the instrument is rotated in the short axis and long axis planes to become positioned in the same plane as the sonography probe. Image created by F Moungondo.

After retrieval of the catheter, the sono-instrument is then introduced through the hole created by the catheter. Initially, the bevel of the instrument is parallel to the skin, but as soon as the sono-instrument is in the sheath, a 90° rotation of the instrument is performed, allowing to turn the bevel of the instrument in the long axis plane, perpendicular to the pulley to be released (Figure 6). It is important to see the whole extremity of the instrument on the sonography screen.

**Figure 7:**
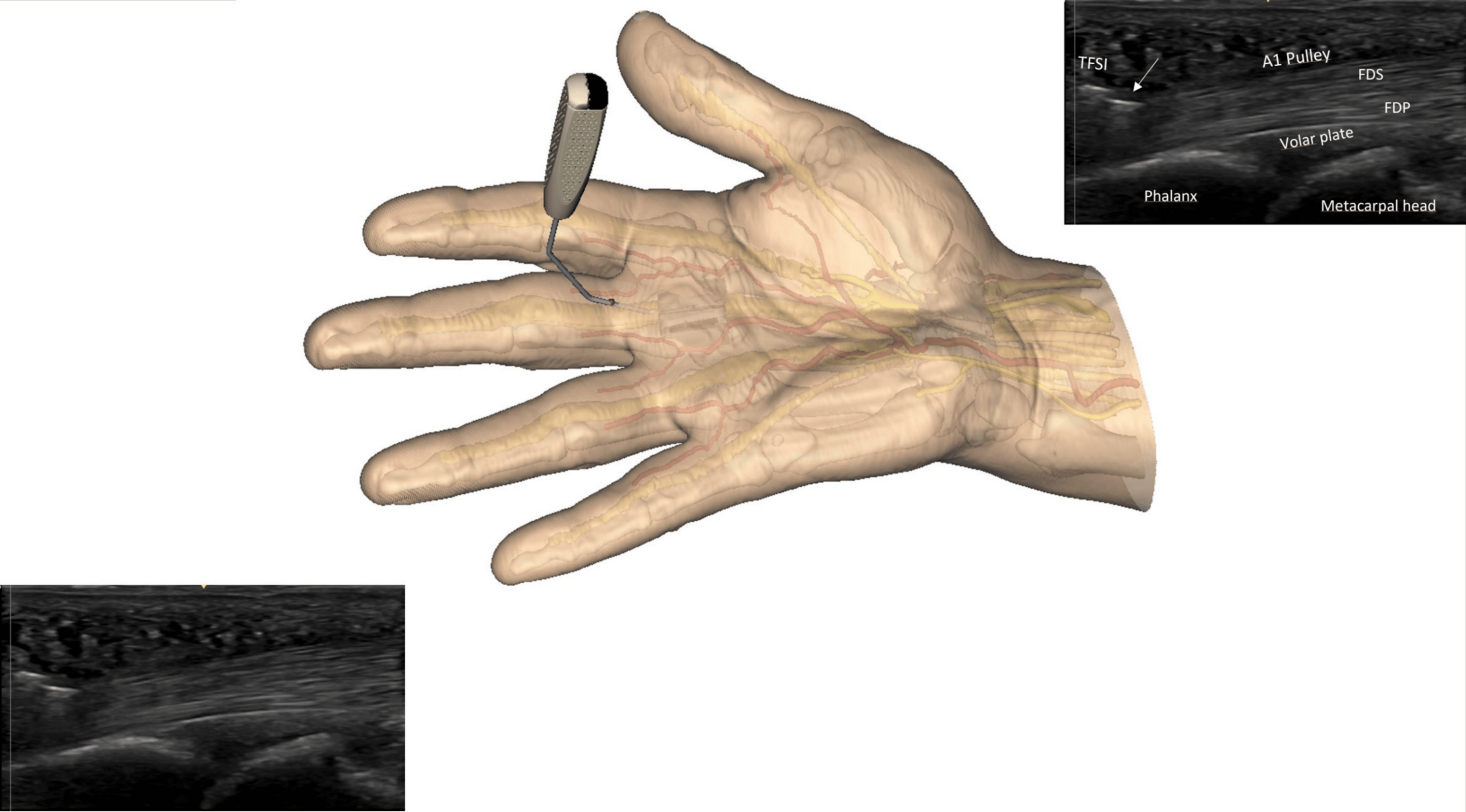
Beginning of the A1 pulley release by using the TF-SI Note the cutting part of the instrument, represented by a straight hyperechoic line on the sonographic image (arrow), thanks to its specific flange. To better understand the sonographic view, the image on the left and bottom is repeated right at the top with a legend. (FDS: Flexor digitorum superficialis; FDP: Flexor digitorum profundus) Note that the sonography probe is intentionally not shown; better observe the cutting instrument underneath the skin. The probe is used throughout the procedure. Image created by F Moungondo.

**Figure 8:**
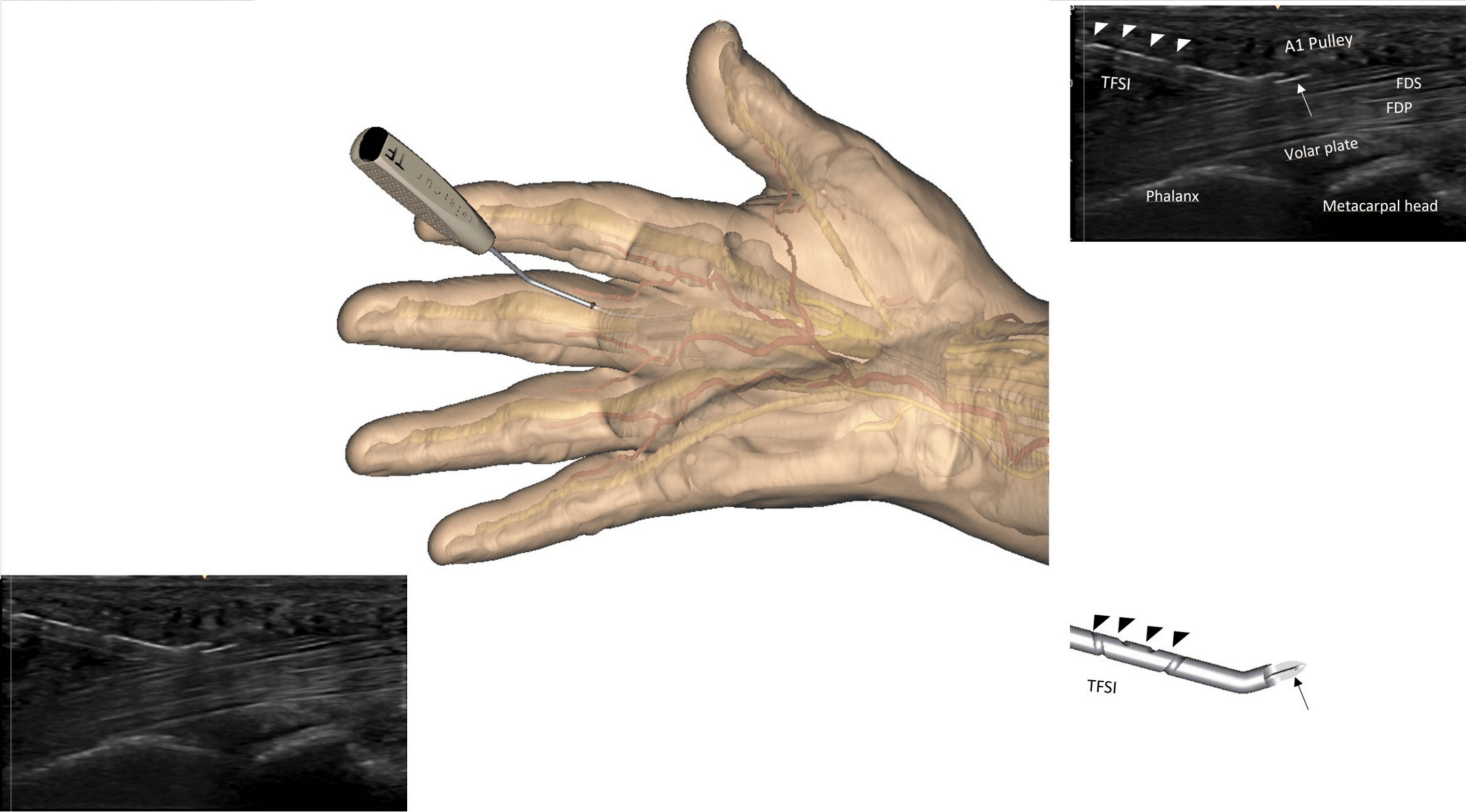
Further advances in the cutting process The whole length of the instrument is seen on the sonography screen. Note the cutting part of the instrument, represented by a straight hyperechoic line on the sonographic image (arrow), thanks to its specific flange. Note the sonography aspect of the spiral groove (arrow heads) that allows you to visualize the axial rotation of the instrument. To better understand the sonographic view, the image on the left and bottom is repeated right at the top with a legend. (FDS: Flexor digitorum superficialis; FDP: Flexor digitorum profundus). Note that the sonography probe is intentionally not shown; better observe the cutting instrument underneath the skin. The probe is used throughout the procedure. Image created by F Moungondo.

The release is progressively performed by crocheting oscillating movements in the long axis plane, from distal to proximal, in a retrograde manner. The center of rotation of the alternating movements corresponds roughly to the entry point. The release is performed in the safe zone, which is in the palmar midline, at the apex of the pulley in the transverse plane, with constant sonographic control of the adjacent neuro-vascular structures (short axis and long axis planes) and of the instrument, highlighted by its sonographic markings (Figures 7, 8). In the thumb, special care is taken to continuously locate the radial collateral digital nerve, which is in some patients at risk when very mobile. The pathway of the nerve must be clearly identified and maintained outside of the plane of cut. Some cracking sounds are usually heard and clearly felt by the patient if the pulley is very thick. The whole release takes a few minutes. As the pulley is close to the skin, the surgeon should avoid too much pressure on the probe, crushing the subcutaneous tissues with a potential risk of an inadvertent cutaneous lesion.

When the release is complete, the patient actively moves the operated finger or thumb, and the surgeon observes clinically and under sonography the disappearance of the triggering phenomenon, the restoration of normal tendon gliding, and the integrity of the flexor tendons. The use of the smooth probe provided by the manufacturer may also be useful to assess the full section of the pulley. If there is still some impingement, the surgeon can reintroduce the sono-instrument for some additional release. No skin closure is needed as there is no incision.

Postoperative Care

At the end of the procedure, if there is any blood oozing at the end of the procedure, compressing the site of the release for five minutes may help to prevent the formation of a hematoma. A compressive dressing is then applied, covering the site of the introduction of the instrument and the site of the release. This dressing is removed 12 hours after the procedure, and no more dressing is then needed. The patient is allowed to wash his or her hands and to resume his or her daily activities the day after the procedure. Passive proximal interphalangeal hyperextension motion exercises are advised for the first three postoperative weeks to prevent or correct a persistent flexion deformity.

Pearls and Pitfalls

The manufacturer provides two models of Sono-Instruments®, one for percutaneous carpal tunnel decompression (CT-SI) and the other for trigger finger release (TF-SI). The surgeon should pay attention not to use the carpal tunnel model for trigger finger surgery.

No tourniquet is used. This allows for good visualization of the pulsating digital arteries, facilitating the localization of the digital nerves. As there is neither a skin incision nor surgical dissection, there is indeed no benefit to using a tourniquet; the installation is quicker, and the operation is more comfortable for the patient.

Care must be taken to ensure that the A1 pulley has been well and completely released. In the case of associated Dupuytren disease with palmar nodes, it might be necessary to also release the A0 pulley, as the patient can present postoperative pain due to persistent flexor tendon impingement under the palmar thickened fascia.

In the thumb, special care is taken throughout the procedure not to injure the radial collateral digital nerve, which can be palpated and sometimes is quite mobile, with the danger of being in the trajectory of the release. However, the nerve is well seen under sonography and can be well protected. Contrarily to other devices, with the TF-SI, the surgeon can perform the release exactly where it needs to be done.

Expected outcomes

Sono-instruments improve surgical performance and patient-related outcomes. The technique offers numerous benefits, including the ability to perform truly percutaneous surgery, avoiding skin incisions and tissue dissection. This is particularly important for patients who require surgery for multiple trigger fingers and who are predisposed to post-operative complications and poor outcomes, like diabetic patients. In such patients, after open release, the rate of skin delayed healing and of wound infection has been reported to be 4.4% and 7.3%, respectively [17]. Even in the absence of skin complications, the palmar skin incision of open or minimally open surgery can cause pain during manual tasks. The sono-instrument technique allows for immediate return to professional and daily living activities in the absence of any incision in the palm or postoperative dressing. Another significant advantage of percutaneous release using sono-instrument, is that it can be performed in an outpatient clinic under local anesthesia, reducing costs for social security/insurances and inconveniences for patients.

Percutaneous release can also be performed using needles. However, multiple punctures are necessary, and associated iatrogenic lesions to the flexor tendons are frequent. The shape and cutting extremity of the sono-instrument device are perfectly adapted to the anatomy of the flexor digital sheath, allowing for safe and effective release without the risk of inadvertent injury to tendons, vessels, or nerves. While other devices may offer faster release times, which is not demonstrated as surgical time is then needed for the installation of the tourniquet, skin incision, and dissection, they also carry a greater risk of iatrogenic injury. The sono-instrument is specifically designed to avoid such risks by allowing surgeons to operate in the safe zone they determine, not the instrument, and this is especially important at the thumb. It is the impression of the authors that, at the thumb, the results of the operation are especially good, possibly because there is only one flexor tendon.

A potential disadvantage of percutaneous treatment is that, as there is no visible scar after a few weeks, there is no visible physical trace of the operation. It happens that patients forget which finger was operated on, and, in the case of a new stenosing tenosynovitis in another finger, they may wrongly believe they will suffer a recurrence. Hence, it is important for surgeons to record in their medical documents exactly which finger(s) was (were) operated on.

Complications

Although we did not encounter in our experience with sono-instrument any iatrogenic complications, incomplete pulley release or lesions to tendons, nerves, or vessels may happen if the surgeon has insufficient experience in hand surgery and in sonography. Surgeons must undergo training and learn hand sonography to master this new form of surgery.

## Discussion

When comparing the open and percutaneous release of the A1 pulley, there are several advantages to the latter. Percutaneous release is well known to be associated with lower complication rates like post-operative pain, infections, and hypertrophic scars. Furthermore, percutaneous release has a faster recovery time and allows an earlier return to activities of daily living [5,6,18]. Another advantage of percutaneous release is that multiple trigger fingers can be done in the same hand at the same time without multiple scars or post-operative stiffness. Saremi et al. compared the outcomes of percutaneous release for trigger fingers using a 19-gauge needle in single-digit involvement versus multiple-digit involvement. They found that percutaneous release was an effective and safe method of treatment, with comparable outcomes for both single and multiple digit involvement, and patients with multiple digit involvement had a longer duration of painkiller use compared to those with single digit involvement, but otherwise the outcomes were similar [19]. In particular, diabetic patients have higher rates of complications after open trigger finger release, and many of them present with multiple trigger fingers. Hypertrophic scars, scar contractures, early sutures falling out, surgical site infections, and digital nerve injury are all documented complications after trigger finger release in diabetics [13]. Employing a truly percutaneous technique using a specially designed instrument like the sono-instrument can prevent nearly all these complications.

Gilberts et al. depicted in their study that patients who underwent percutaneous trigger finger release had less postoperative pain and were able to return to work at an earlier time. They also compared open vs. percutaneous release and found that the percutaneous technique leads to a greater range of motion, especially in the first week postoperatively [20].

One of the concerns of percutaneous technique is the incomplete release of the A1 pulley and injury to the surrounding neurovascular structures [12]. Wang et al., in their meta-analysis, demonstrated that there is no difference in complication and recurrence rates between open and percutaneous techniques [18]. Slesarenko et al. reported in their cadaveric study a 59% success rate of percutaneous release using an 18-gauge needle [11]. This low success rate may be attributable to the blind technique. Cromheecke et al. reported in their series a success rate of 98.7% with percutaneous ultrasound-guided trigger finger release using a second-generation knife [13]. The sono-instrument technique requires the utilization of ultrasound during the whole procedure, thereby ensuring a complete release of the A1 pulley at the end of the procedure. The release is performed under local anesthesia, and by that patients are asked at the end of the procedure to flex and extend their surgical digit, which allows us to further assess if the release of the A1 pulley is well complete [14].

The TF-SI overcomes some of the limitations of the other medical devices by allowing for true percutaneous surgery. The absence of a skin incision at the palm is advantageous, in particular for manual workers and diabetics. Without a skin incision, the patient can also wash his/her hand the next day. It is possible to treat multiple trigger fingers at the same time or to perform bilateral surgery. As no other surgical instrument is needed, nor is a tourniquet, the sono-instrument is particularly well adapted to office surgery. Finally, because no retraction of the tissues is needed, a single operator can perform the procedure without surgical assistance. An assistant is, however, needed to trigger the thumb and hold the digit. We have evaluated the Sono-Instruments® for treating carpal tunnel syndrome and trigger finger in 30 patients and found them to be safe and effective, with all patients successfully treated and most returning to daily activities and work within a week. Significant functional improvements were noted at two months. The study concluded that Sono-Instruments® offers a quick functional recovery for these conditions, with a level II evidence rating [15].

## Conclusions

In conclusion, this technical article presents a detailed technique for the use of the newly designed sono-instrument for trigger finger treatment. Its small size, with a 1.5-mm diameter, allows the entire procedure to be conducted through a simple puncture, affording the surgeon precise control over the incision site. This feature potentially enhances safety compared to the direct cuts of traditional instruments. Moreover, the sono-instrument is designed to release the pulley effectively without being excessively sharp, thereby minimizing the risk of accidental injury to surrounding structures like tendons, vessels, or nerves. Its unique design includes lateral flanges and a spiral groove on the rod, which not only improve ultrasound visibility but also facilitate rotatory orientation. This technique implies a simple learning curve and can be performed under local anesthesia in an outpatient clinical setting, reducing discomfort for patients and costs for insurance/social security.

## References

[1] Gil JA, Hresko AM, Weiss AC (2020). Current concepts in the management of trigger finger in adults. J Am Acad Orthop Surg.

[2] Sampson SP, Badalamente MA, Hurst LC Pathobiology of the human A1 pulley in trigger finger. Jr Han Surg.

[3] Bonnici AV, Spencer JD (1988). A survey of 'trigger finger' in adults. J Hand Surg Br.

[4] Urbaniak JR, Roth JH (1982). Office diagnosis and treatment of hand pain. Ortho Clin Nor Ame.

[5] Dierks U, Hoffmann R, Meek MF (2008). Open versus percutaneous release of the A1-pulley for stenosing tendovaginitis: a prospective randomized trial. Tech Hand Up Extrem Surg.

[6] Paulius KL, Maguina P (2009). Ultrasound-assisted percutaneous trigger finger release: is it safe?. Hand (N Y).

[7] Hopkins J, Sampson M (2014). Percutaneous tenotomy: Development of a novel, percutaneous, ultrasound-guided needle-cutting technique for division of tendons and other connective tissue structures. J Med Imaging Radiat Oncol.

[8] Lapègue F, André A, Meyrignac O (2016). US-guided Percutaneous Release of the Trigger Finger by Using a 21-gauge Needle: A Prospective Study of 60 Cases. Radiology.

[9] Rajeswaran G, Lee JC, Eckersley R, Katsarma E, Healy JC (2009). Ultrasound-guided percutaneous release of the annular pulley in trigger digit. Eur Radiol.

[10] Guo D, Guo D, Guo J, McCool LC, Tonkin B (2018). A Cadaveric Study of the Thread Trigger Finger Release : The First Annular Pulley Transection Through Thread Transecting Technique. Hand (N Y).

[11] Slesarenko YA, Mallo G, Hurst LC, Sampson SP, Serra-Hsu F (2006). Percutaneous release of A1 pulley. Tech Hand Up Extrem Surg.

[12] Chern TC, Jou IM, Yen SH, Lai KA, Shao CJ (2005). Cadaveric study of sonographically assisted percutaneous release of the A1 pulley. Plast Reconstr Surg.

[13] Cromheecke M, Haignère V, Mares O, De Keyzer PB, Louis P, Cognet JM (2022). An Ultrasound-guided percutaneous surgical technique for trigger finger release using a minimally invasive surgical knife. Tech Hand Up Extrem Surg.

[14] (2023). Spirecut: Trigger Finger Sono-Instrument®. https://spirecut.com/trigger-finger-sono-instrument-tf/.

[15] Moungondo F, Van Rompaey H, Schuind F Prospective evaluation of a novel device for ultrasound-guided percutaneous treatment of carpal tunnel and trigger finger disease. Efficacy and safety of sono-instruments®. Jr Han Sur.

[16] Guerini H, Pessis E, Theumann N (2008). Sonographic appearance of trigger fingers. J Ultrasound Med.

[17] Federer AE, Baumgartner RE, Cunningham DJ, Mithani SK (2020). Increased Rate of Complications following Trigger Finger Release in Diabetic Patients. Plast Reconstr Surg.

[18] Wang J, Zhao JG, Liang CC (2013). Percutaneous release, open surgery, or corticosteroid injection, which is the best treatment method for trigger digits?. Clin Orthop Relat Res.

[19] Saremi H, Hakhamaneshi E, Rabiei MA (2016). Percutaneous release of trigger fingers: comparing multiple digits with single digit involvement. Arch Bone Jt Surg.

[20] Gilberts EC, Beekman WH, Stevens HJ, Wereldsma JC (2001). Prospective randomized trial of open versus percutaneous surgery for trigger digits. J Hand Surg Am.

