# Correction: Retrograde Percutaneous Release of Trigger Finger or Thumb Using Sono-Instruments®: Detailed Technique, Pearls, and Pitfalls

**DOI:** 10.7759/cureus.c287

**Published:** 2025-09-09

**Authors:** Fabian Moungondo, Luc Van Ovestraeten, Mohammad O Boushnak, Frédéric Schuind

**Affiliations:** 1 Department of Orthopedics and Traumatology, Université Libre de Bruxelles, Erasme University Hospital, Brussels, BEL; 2 Department of Orthopaedics and Traumatology, Hand and Wrist Center, Hand and Foot Surgery Unit (HFSU), AO Foundation, Erasme University Hospital, Tournai, BEL; 3 Department of Orthopedics and Sports Medicine, North Sydney Orthopaedic and Sports Medicine Centre, Mater Hospital, Sydney, AUS

The Ethics Statement and Conflict of Interest Disclosures section of this article has been corrected to indicate that this study did involve human subjects.

